# Predictors of perceived risk in first-degree relatives of patients with rheumatoid arthritis

**DOI:** 10.1136/rmdopen-2022-002606

**Published:** 2022-12-05

**Authors:** Sarah Bunnewell, Imogen Wells, Dawit Zemedikun, Gwenda Simons, Christian D Mallen, Karim Raza, Marie Falahee

**Affiliations:** 1 Department of Rheumatology, Sandwell and West Birmingham NHS Trust, Birmingham, UK; 2 Rheumatology Research Group, Institute of Inflammation and Ageing, University of Birmingham, Birmingham, UK; 3 Institute of Applied Health Research, University of Birmingham, Birmingham, UK; 4 Primary Care Centre Versus Arthritis, School of Medicine, Keele University, Keele, UK; 5 NIHR Birmingham Biomedical Research Centre, University Hospitals Birmingham NHS Foundation Trust and University of Birmingham, Birmingham, UK; 6 MRC Versus Arthritis Centre for Musculoskeletal Ageing Research and the Research into Inflammatory Arthritis Centre Versus Arthritis, University of Birmingham, Birmingham, UK

**Keywords:** Arthritis, Rheumatoid, Patient Reported Outcome Measures, Psychology

## Abstract

**Objectives:**

To define variables associated with perceived risk of developing rheumatoid arthritis (RA) in first-degree relatives (FDRs) of patients with RA.

**Methods:**

Patients with RA and their FDRs were invited to complete cross-sectional surveys. FDR and index patient responses were linked. FDRs’ perceived absolute risk, comparative risk, experiential risk and worry about risk were assessed using 5-point Likert scales. FDR predictor variables included demographics, illness perceptions and psychosocial variables. Patient predictors of FDR perceived risk were assessed. Binary logistic regression examined the relationship between FDR characteristics and perceived risk of RA. Generalised estimating equations assessed whether patient variables predicted FDR’s perceived risk.

**Results:**

396 FDRs returned a survey. 395 FDRs provided sufficient data and were included in analysis. Paired data from 213 patients were available for 291 of these FDRs. All measures of perceived risk were inter-correlated. 65.2% of FDRs perceived themselves to be ‘likely’ or ‘very likely’ to develop RA in their lifetime. Relationship with index patient, high health anxiety, female gender, long perceived RA duration, high perceived concern about RA, negative perceived emotional impact of RA and low perceptions of how well treatment would control RA were all associated with increased FDRs’ perceived risk. Patient characteristics did not associate with FDRs’ risk perceptions.

**Conclusions:**

FDRs’ perceived risk of RA was high. Key predictors included being a child of a patient with RA, higher health anxiety and lower perceptions of RA treatment control. An understanding of these predictors will inform the development of tailored risk communication resources and preventive clinical strategies for RA.

**WHAT IS ALREADY KNOWN ON THIS TOPIC**

- Risk of rheumatoid arthritis (RA) is three to five times higher in first-degree relatives (FDRs) and there is increasing interest in predictive and preventive approaches for this group. Perceptions about risk (including the extent to which patients think they are at risk of a condition) predict health behaviours, including interest in predictive testing for RA and preferences for preventive treatment of RA.

**WHAT THIS STUDY ADDS**

- 65.2% of FDRs perceived themselves to be ‘likely’ or ‘very likely’ to develop RA. Key predictors of perceived risk were identified, and included domains of the Brief Illness Perceptions Questionnaire.

**HOW THIS STUDY MIGHT AFFECT RESEARCH, PRACTICE OR POLICY**

- This study highlights the need to develop effective risk communication resources for those at risk of RA that address key perceptual variations around RA.

## Introduction

Rheumatoid arthritis (RA) is a chronic destructive polyarthritis which affects approximately 1% of the population.^1^ It is associated with disability, reduced life expectancy, and significant societal and healthcare costs.^2^ There is increasing interest in the identification of individuals at risk of developing RA^3^ and in early intervention to reduce this risk.^4^

Multiple environmental and genetic factors are known to influence the risk of developing RA. Examples of environmental factors include smoking, dietary factors and occupational pollutant exposure.^5^ Genetic factors play a significant role in RA risk. Risk is approximately three to five times higher in those with a family history of RA, most prominently in first-degree relatives (FDRs).^1^ Consequently, efforts are being made to develop and test predictive algorithms and preventative interventions in this at-risk group.^6^ For these efforts to be effective, it is important to understand the views and receptiveness of these individuals. Recent evidence suggests that the majority of FDRs are interested in predictive testing^7^ and preventive treatment.^8^ Factors that increased interest included information seeking preferences, positive attitudes towards risk knowledge, perceived risk and regular contact with their relative with RA.^7^ As perceived risk predicted interest in predictive testing, it is important to understand to what extent and why people feel at risk.

Risk perception is a key element of several health behaviour theories.^9^ The Health Belief Model^10^ has been extensively used to predict engagement in risk prevention and reduction behaviours, and suggests that health behaviours can be influenced by perceived susceptibility to and severity of disease and the benefits of and barriers to change. Therefore, understanding the relationships between perception of risk and illness beliefs and other patient characteristics is important in the development of informational tools that form part of prevention strategies.

Variables associated with perceived risk have been explored in other diseases including breast, gastric and prostate cancers, cardiovascular disease and type 2 diabetes.^11–15^ While findings are not uniform between studies or diseases, there are a variety of factors which have demonstrated significant associations with perceived risk across multiple diseases, including age, family history, education level, relationship to index patient, perceived severity and worry about risk of developing the disease.

This study assesses the relationships between FDRs’ perceptions regarding their risk of RA, and their illness beliefs, demographic and psychosocial variables. The impact of patient variables on FDRs’ risk perception is also assessed.

## Methods

### Design

Both patients with RA and their FDRs completed cross-sectional surveys, which explored interest in predictive testing, demographics, illness beliefs and psychosocial variables.^7^ The current paper represents a secondary analysis of FDR responses with the objective of defining variables that are predictive of risk perception in FDRs of patients with RA.

### Participants and setting

Patients with a confirmed diagnosis of RA were invited by a rheumatologist or research nurse to participate via rheumatology outpatient clinics in the West Midlands, UK, between March 2017 and January 2020. Patients were provided with a survey pack containing printed surveys for themselves and two FDRs. Additional FDR surveys could be requested if required. FDRs were able to participate even if the patient with RA did not wish to themselves. Surveys were uniquely coded to allow linkage of returned surveys between individual patients and their cognate FDRs. FDRs were eligible if they (A) were biological children or full siblings of a patient with a confirmed diagnosis of RA; (B) were aged 18 years or over; (C) did not have a diagnosis of RA and (D) were able to complete the printed survey in English and indicate consent by completing a series of checkboxes to indicate that they agreed to take part.

### Outcome measures

Measures used are outlined below, further details of why these measures were chosen and the internal reliability and test–retest reliability are described in a previous paper.^7^

#### Perceived risk of RA

Risk perceptions were assessed using four items; absolute risk, comparative risk, experiential risk and worry about risk. Each item was measured using a 5-point Likert-type response scale with higher scores indicating higher perceived risk. These risk measures were chosen as, although moderately correlated, evidence suggests that they capture different constructs and may have differing associations with risk evaluation^16^ and health behaviour change.^17 18^ Absolute risk assesses overall perception of the likelihood that the individual will develop RA in their lifetime. Comparative risk assesses the individual’s perception of the likelihood that they will develop RA in their lifetime compared with other people of the same demographic. Experiential risk assesses the extent to which the individual agrees that they feel at risk of developing RA in their lifetime. Worry about risk assesses the extent to which the individual is worried about getting RA in their lifetime. The specific statements used in the survey to assess risk measures are outlined separately below.

##### Perceived absolute risk

Assessed using the statement ‘How likely do you think it is that you will develop rheumatoid arthritis in your lifetime?’. Options ranged from ‘very unlikely’ to ‘very likely’.

##### Perceived comparative risk

Assessed using the statement ‘Compared with other people your age, gender and race, how likely do you think it is that you will develop rheumatoid arthritis in your lifetime?’. Options ranged from ‘much less likely’ to ‘much more likely’.

##### Perceived experiential risk

Assessed using the statement ‘I feel that I am at risk of getting rheumatoid arthritis in my lifetime’. Options ranged from ‘strongly disagree’ to ‘strongly agree’.

##### Worry about risk

Assessed using the statement ‘I am worried about getting rheumatoid arthritis in my lifetime’. Options ranged from ‘strongly disagree’ to ‘strongly agree’. Worry is correlated with perceived risk across multiple diseases and the two are also thought to interact to influence health behaviours.^9^ Conversely, some theorise that worry refers to emotional reactions rather than intellectual judgement and thus should be considered separately to risk perception.^19^

### Predictor variables

#### Illness perceptions

Illness perceptions of RA were measured using a modified version of the Brief Illness Perceptions Questionnaire (Brief IPQ).^20^ The IPQ assesses beliefs about the disease in eight domains: consequences, timeline, personal control, treatment control, identity, concern, understanding and emotion. The wording of items was modified to be applicable for at-risk individuals,^21^ for example, ‘If you were to develop rheumatoid arthritis, how much do you think it would affect your life?’. A higher score on the 11-point scale for items 1, 2, 5, 6 and 8, and a lower score in items 3, 4 and 7 indicated a more threatening view of RA. An overall score was calculated by reverse scoring items 3, 4 and 7 and adding these to the remaining items. A higher summed score indicated a more threatening view of RA.

#### Demographic variables

Demographic variables included gender, age, ethnicity, employment status, the highest level of education and smoking status. Postcodes were used to calculate the Multiple Deprivation Index with scores between 1 and 10 (1 indicating most deprived area, 10 indicating least deprived area).

#### Relationship with index patient

The FDRs also reported their relationship to their index patient (child or sibling), whether they lived with the index patient at the time of survey and their frequency of communication with patient (measured using 4-point scale ranging ‘never’ to ‘every day’).

#### Understanding of written and numerical information

FDR’s reported ability to understand numerical information was assessed using the three-item Subjective Numeracy Score.^22^ Each item was scored on a 6-point scale (total score ranging 3–18) with a higher overall score indicated a stronger perceived mathematical ability.

Health literacy was assessed using the Single Item Literacy Screener. On a 5-point scale, responses ranged from 0 (‘never’) to 4 (‘always’), with a score above 2 indicating some difficulty reading health-related material.^23^

#### Coping styles

The Brief Approach/Avoidance Coping Questionnaire assessed coping styles in stressful situations in three domains: cognitive, socioemotional and action-related.^24^ The questionnaire consisted of 12 items, each measured on a 5-point scale ranging from 0 (‘strongly disagree’) to 4 (‘strongly agree’). Total scores ranged 0–48 with higher scores indicating higher approach/lower avoidance coping styles.

#### Dispositional optimism

Three items from the Life Orientation Test-Revised were used to assess dispositional optimism. Each item was measured on 5-point scale ranging from 0 (‘strongly disagree’) to 4 (‘strongly agree’). Total score ranged from 0 to 12 with higher scores indicating increased optimism.^25^

#### Health anxiety

Health anxiety was measured using The Short Health Anxiety Inventory.^26^ Eighteen items assessed FDRs’ worry about health, awareness of bodily sensations and changes and feared consequence of illness. For each item, participants selected one of four statements that they felt best reflected their feelings in the previous 6 months. Total scores ranged from 0 to 54 with scores above 27 indicating health anxiety.

#### Index patient measures

Data were also collected from index patients with RA and linked to FDRs for analysis. Measures included age, gender, time since RA diagnosis, RA treatment and RA disease impact measured using the Rheumatoid Arthritis Impact of Disease (RAID) scale.^27^ Seven items assessed the extent to which the index patient’s RA impacted the following domains in the preceding week: pain, ability, fatigue, sleep, physical well-being, emotional well-being and coping. Domains were measured using an 11-point scale with 0 indicating no impact and 10 indicating extreme impact. Scores were weighted and summed; total scores ranged between 0 and 10 with higher scores representing worse reported disease status.^27^

### Analysis

Statistical analyses were conducted using IBM SPSS Statistics V.27.0.

#### Association between FDR characteristics and perceived risk

Demographic and psychosocial characteristics were summarised using descriptive statistics. Responses to items assessing perceived absolute risk, comparative risk, experiential risk and worry about risk, were dichotomised into ‘low’ and ‘high’. FDR’s were classified as high perceived absolute risk if they perceived themselves to be either ‘very likely’ or ‘likely’ to develop RA in their lifetime. The same threshold for dichotomising variables was used for the equivalent responses to comparative risk, experiential risk and worry about risk questions with the top two responses on the 5-point Likert scale being taken to indicate high perceived risk/high levels of worry about risk.

The univariate associations between continuous, categorical and ordinal variables and FDR’s perceived risk were assessed using independent samples t-tests, χ^2^ tests and Mann-Whitney U tests, respectively. Predictor variables that were significant, using a significance level <0.05, were carried forward to create a binary logistic regression model. A backwards stepwise approach was used with a default cut-off p value of 0.1.

#### Association between patient characteristics and FDRs’ perceived risk

The demographic and clinical characteristics of patients were described using descriptive statistics. To allow for the possible non-independence of multiple FDRs paired with a single patient, generalised estimating equations using an exchangeable working correlation matrix assessed whether patient characteristics could predict their FDR’s perceived risk.

### Patient and public involvement

Three patient research partners (PRPs) contributed to the design of this study during a group meeting and via email. They advised on study recruitment, highlighting that FDRs or patients who had not previously considered that they, or their relative, may have increased risk status, might experience anxiety related to the issues raised in the surveys. Consequently, patients were invited to take part in the outpatient clinic setting by members of the healthcare team who were able to address any concerns. In addition, as a part of the debriefing letter, an information resource about RA risk in family members of patients developed by a multidisciplinary team of clinicians, scientists and patient partners for a previous project (https://cordis.europa.eu/project/id/305549/reporting) and sources of further support were provided at the end of the survey. Patients with a recent diagnosis (within the previous 6 months) were not approached as PRPs suggested this might add to any anxiety surrounding new diagnosis and treatment.

The design and content of the survey was reviewed by PRPs. Examples of revisions made in response to their input included the use of a subjective rather than objective numeracy measure, inclusion of a table of contents to inform participants of the nature of the questions prior to responding and, finally, free-text sections to provide an opportunity for open-ended responses.

## Results

Of 1720 patients who were provided with a survey pack, 396 eligible FDRs returned a survey. One FDR was excluded from analyses as responses were missing for all of the outcome variables. A second FDR responded to two of the four outcome variable measures and thus was included in analysis. All other FDRs had complete outcome variable data. In total, data from 395 FDRs were included in analysis.

Paired data from 213 patients were available for 291 of these FDRs. Some FDRs did not have a linked patient survey and in other cases, multiple FDRs were linked to a single patient survey. Two patients had four FDRs, eight had three FDRs, 56 had two FDRs and the remaining 147 patients had one FDR.

### FDR’s perceived risk

The distributions of perceived risk/worry outcome variables are described in table 1. For further analyses, data were dichotomised into two groups. 65.2% of FDRs perceived themselves to be ‘likely’ or ‘very Likely’ to develop RA in their lifetime (perceived absolute risk).

**Table 1:** Distribution of perceived risk

Category of perceived risk/worry	No of FDRs (percentage (%))			
	Absolute risk N=394*	Relative risk N=394*	Experiential risk N=395	Worry about risk N=395
1 (low)	5 (1.3)	6 (1.5)	3 (0.8)	12 (3.0)
2	31 (7.9)	17 (4.3)	28 (7.1)	42 (10.6)
3	101 (25.6)	155 (39.3)	92 (23.3)	116 (29.4)
4	202 (51.3)	174 (44.2)	211 (53.4)	166 (42.0)
5 (high)	55 (13.9)	42 (10.6)	61 (15.4)	59 (14.9)

*N=1 missing (0.3%).

FDRs, first-degree relatives.

All measures of FDRs’ perceived risk of developing RA were highly intercorrelated, ranging from r(392)=0.80, p<0.001 (absolute risk and experiential risk) to r(392)=0.48, p<0.001 (comparative risk and worry about risk). Online supplemental figure 1 illustrates the correlations between the four outcome variables.

10.1136/rmdopen-2022-002606.supp1 Supplementary data

The demographic and psychosocial characteristics of FDRs and their univariate associations with perceived risk are summarised in table 2. Age, relationship to index patient, IPQ personal control and treatment control subscales and health anxiety were significantly associated with perceived absolute risk. Age, relationship to index patient, frequency of communication, health anxiety and five domains from the IPQ were significantly associated with perceived relative risk. Relationship to index patient, frequency of communication, health literacy, health anxiety and five domains from the IPQ were significantly associated with perceived experiential risk. Female gender, relationship to index patient, frequency of communication, subjective numeracy, health anxiety and six domains from the IPQ were significantly associated with worry about risk.

**Table 2:** FDR descriptive statistics and univariate analysis

Characteristic of FDR	All Patients n=395	Perceived absolute risk			Perceived relative risk			Perceived experiential risk			Worry about risk		
		Low n=137	High n=257	P value	Low n=178	High n=216	P value	Low n=123	High n=272	P value	Low n=170	High n=225	P value
Age, years													
	42.3 (14.3)	44.8 (15.9)	41.0 (13.2)	0.02	44.0 (15.7)	41.0 (12.9)	<0.001	45.5 (16.1)	40.9 (13.2)	0.07	43.7 (15.6)	41.3 (13.2)	0.11
n=16 missing; mean (SD); T													
Gender													
Male	136 (35.0)	45 (33.1)	91 (66.9)	0.55	65 (47.8)	71 (52.2)	0.43	43 (31.6)	93 (68.4)	0.87	73 (53.7)	63 (46.3)	0.002
Female	253 (65.0)	91 (36.1)	161 (63.9)		110 (43.7)	142 (56.3)		78 (30.8)	175 (69.2)		94 (37.2)	159 (62.8)	
n=6 missing; frequency (%); CS													
Ethnic group													
White	327 (83.2)	114 (34.9)	213 (65.1)	0.13	154 (47.1)	173 (52.9)	0.17	103 (31.5)	224 (68.5)	0.57	142 (43.4)	185 (56.6)	0.3
Asian	36 (9.2)	12 (33.3)	24 (66.7)		10 (27.8)	26 (72.2)		10 (27.8)	26 (72.2)		11 (30.6)	25 (69.4)	
Black	14 (3.6)	1 (7.7)	12 (92.3)		5 (38.5)	8 (61.5)		3 (21.4)	11 (78.6)		8 (57.1)	6 (42.9)	
Mixed	15 (3.8)	8 (53.3)	7 (46.7)		8 (53.3)	7 (46.7)		7 (46.7)	8 (53.3)		8 (53.3)	7 (46.7)	
Other	1 (0.3)	0 (0.0)	1 (100.0)		0 (0.0)	1 (100.0)		0 (0.0)	1 (100.0)		0 (0.0)	1 (100.0)	
n=2 missing; frequency (%); CS													
Deprivation Index													
	4 (2–7)	5 (2–8)	4 (2–7)	0.13	4 (2–7)	5 (2–7)	0.92	4 (2–7)	5 (2–7)	0.08	5 (2–8)	4 (2–7)	0.13
n=82 missing; median (IQR); U													
Employment status													
Employed	297 (76.3)	99 (33.3)	198 (66.7)	0.17	132 (44.4)	165 (55.6)	0.22	86 (29.0)	211 (71.0)	0.1	124 (41.8)	173 (58.2)	0.3
Unemployed	61 (15.7)	28 (45.9)	33 (54.1)		34 (55.7)	27 (44.3)		26 (42.6)	35 (57.4)		29 (47.5)	32 (52.5)	
Other	31 (8.0)	10 (33.3)	20 (66.7)		12 (40.0)	18 (60.0)		11 (35.5)	20 (64.5)		17 (54.8)	14 (45.2)	
n=6 missing; frequency (%); CS													
Education level													
A-level or lower	186 (49.2)	68 (36.8)	117 (63.2)	0.56	91 (49.2)	94 (50.8)	0.21	68 (36.6)	118 (63.4)	0.05	80 (43.0)	106 (57.0)	0.81
Higher than A-level	192 (50.8)	65 (33.9)	127 (66.1)		82 (42.7)	110 (57.3)		52 (27.1)	140 (72.9)		85 (44.3)	107 (55.7)	
n=17 missing; frequency (%); CS													
Smoking status													
Current smoker	40 (10.3)	10 (25.0)	30 (75.0)	0.33	15 (37.5)	25 (62.5)	0.36	11 (27.5)	29 (72.5)	0.45	15 (37.5)	25 (62.5)	0.7
Ex-smoker	110 (28.4)	41 (37.6)	68 (62.4)		55 (50.5)	54 (49.5)		40 (36.4)	70 (63.6)		48 (43.6)	62 (56.4)	
Never smoker	237 (61.2)	86 (36.3)	151 (63.7)		108 (45.6)	129 (54.4)		72 (30.4)	165 (69.6)		106 (44.7)	131 (55.3)	
n=8 missing; frequency (%); CS													
Relationship to index patient													
Child	295 (75.4)	86 (29.3)	208 (70.7)	<0.001	113 (38.4)	181 (61.6)	<0.001	72 (24.4)	223 (75.6)	<0.001	115 (39.0)	180 (61.0)	0.002
Sibling	96 (24.6)	51 (53.1)	45 (46.9)		65 (67.7)	31 (32.3)		51 (53.1)	45 (46.9)		55 (57.3)	41 (42.7)	
n=4 missing; frequency (%); CS													
Living in same household as index patient													
Yes	77 (19.6)	27 (35.1)	50 (64.9)	0.94	38 (49.4)	39 (50.6)	0.38	22 (28.6)	55 (71.4)	0.6	37 (48.1)	40 (51.9)	0.29
No	316 (80.4)	109 (34.6)	206 (65.4)		138 (43.8)	177 (56.2)		100 (31.6)	216 (68.4)		131 (41.5)	185 (58.5)	
n=2 missing; frequency (%); CS													
Frequency of communication with index patient													
Never	0 (0.0)	0 (0.0)	0 (0.0)	0.07	0 (0.0)	0 (0.0)	0.03	0 (0.0)	0 (0.0)	0.04	0 (0.0)	0 (0.0)	0.002
Rarely	4 (1.0)	1 (25.0)	3 (75.0)		4 100.0)	0 (0.0)		2 (50.0)	2 (50.0)		2 (50.0)	2 (50.0)	
Sometimes	20 (5.1)	12 (60.0)	8 (40.0)		12 (60.0)	8 (40.0)		9 (45.0)	11 (55.0)		13 (65.0)	7 (35.0)	
Often	153 (39.1)	56 (36.6)	97 (63.4)		77 (50.3)	76 (49.7)		53 (34.6)	100 (65.4)		75 (49.0)	78 (51.0)	
Daily	214 (54.7)	67 (31.5)	146 (68.5)		83 (39.0)	130 (61.0)		58 (27.1)	156 (72.9)		78 (36.4)	136 (63.6)	
n=4 missing; frequency (%); U													
Subjective numeracy													
	15 (11–17)	15 (13–17)	15 (11–18)	0.48	15 (11–17)	15 (12–18)	0.57	15 (12–17)	15 (11–18)	0.74	15 (13–18)	14 (11–17)	0.01
n=4 missing; median (IQR); U													
Health literacy													
	0 (0–0)	0 (0–0)	0 (0–0)	0.11	0 (0–0)	0 (0–0)	0.34	0 (0–0)	0 (0–0)	0.04	0 (0–0)	0 (0–0)	0.52
n=4 missing; median (IQR); U													
Illness perceptions													
Consequences n=4 missing	8 (7–9)	8 (7–9)	8 (7–9)	0.24	8 (7–9)	8 (7–9)	0.03	8 (6–9)	8 (7–9)	0.04	7 (6–9)	8 (7–9)	<0.001
Timeline n=5 missing	10 (9–10)	10 (9–10)	10 (9–10)	0.11	10 (9–10)	10 (10–10)	0.006	10 (9–10)	10 (9–10)	0.049	10 (9–10)	10 (10–10)	0.02
Personal control n=5 missing	5 (3–7)	5 (3–7)	5 (3–6)	0.04	5 (3–7)	5 (3–6)	0.3	5 (3–7)	5 (3–6)	0.02	5 (3–7)	5 (3–6)	0.03
Treatment control n=5 missing	7 (5–8)	7 (5–8)	6 (5–8)	0.002	7 (5–8)	6 (5–8)	0.004	7 (5–9)	6 (5–8)	<0.001	7 (5–8)	6 (5–8)	<0.001
Identity n=4 missing	8 (7–8)	7 (7–8)	8 (7–8)	0.15	7 (6–8)	8 (7–8)	0.02	7 (6–8)	7 (7–8)	0.08	7 (6–8)	8 (7–9)	<0.001
Concern n=2 missing	8 (7–10)	8 (7–10)	8 (7–10)	0.12	8 (7–10)	8.5 (7–10)	0.04	8 (7–10)	8 (7–10)	0.07	8 (7–9)	9 (8–10)	<0.001
Understanding n=2 missing	7 (6–9)	8 6–9)	7 (6–9)	0.4	7 (6–9)	7 (6–9)	0.66	7 (6–9)	7 (6–9)	0.55	8 (6–9)	7 (6–9)	0.32
Emotion n=2 missing	7 (6–9)	7 (6–9)	7 (6–9)	0.79	7 (6–9)	7 (6–9)	0.06	7 (5–8)	7 (6–9)	0.02	7 (5–8)	8 (7–9)	<0.001
Median (IQR); U													
Coping style													
	30.1 (5.5)	30.0 (5.5)	30.1 (5.5)	0.84	30.3 (5.5)	29.9 (5.4)	0.86	30.5 (5.4)	29.9 (5.5)	0.34	30.2 (5.6)	30.0 (5.4)	0.64
n=8 missing; mean (SD); T													
Optimism													
	7.3 (2.3)	7.4 (2.3)	7.3 (2.3)	0.81	7.4 (2.4)	7.2 (2.3)	0.64	7.5 (2.1)	7.2 (2.4)	0.26	73 (2.4)	7.3 (2.2)	0.87
n=5 missing; mean (SD); T													
Health anxiety													
	12 (8–18)	10 (7–15)	13 (9–18)	<0.001	10 (7–16)	14 (9–18)	<0.001	10 (6–13)	13.5 (9–18)	<0.001	9 (6–14)	14 (9–18)	<0.001

n=17 missing; Median (IQR); U.

CS, χ^2^ test; FDR, first-degree relative; T, independent samples t-test; U, Mann-Whitney U test.

Backwards stepwise logistic regression was performed on the five significant variables for perceived absolute risk and a final model with three variables was identified (table 3). The model was statistically significant χ^2^(3) = 32.458, p<0.001 and explained 11.6% (Nagelkerke R^2^) of the variance in perceived absolute risk of RA. It correctly classified 65.4% of cases. Children were almost three times more likely to perceive themselves at high risk compared with siblings. With each one unit increase on the IPQ treatment control scale there was a 11% decrease in likelihood of perceiving oneself at high risk. Higher health anxiety scores were associated with increased likelihood of high perceived risk.

**Table 3:** ORs for perceived risk of developing RA in FDRs of patients with RA in multivariable analysis

FDR characteristic	Perceived absolute risk		Perceived comparative risk		Perceived experiential risk		Worry about risk	
	OR (95% CI)	P value	OR (95% CI)	P value	OR (95% CI)	P value	OR (95% CI)	P value
Gender								
Male*	–	–	–	–	–	–	–	–
Female	–	–	–	–	–	–	1.98 (1.19 to 3.27)	0.01
Relationship to index patient								
Sibling*	–	–	–	–	–	–	–	–
Child	2.80 (1.70 to 4.61)	<0.001	3.43 (2.04 to 5.78)	<0.001	3.89 (2.24 to 6.75)	<0.001	2.26 (1.30 to 3.94)	0.004
Health literacy	–	–	–	–	1.50 (1.00 to 2.25)	0.05	–	–
Illness perceptions								
Timeline	–	–	1.25 (1.05 to 1.48)	0.01	1.17 (0.99 to 1.38)	0.07	–	–
Treatment control	0.89 (0.79 to 0.99)	0.03	0.91 (0.81 to 1.01)	0.06	0.87 (0.77 to 0.98)	0.02	0.84 (0.74 to 0.94)	0.004
Concern	–	–	–	–	–	–	1.36 (1.13 to 1.63)	0.001
Emotion	–	–	–	–	–	–	1.20 (1.02 to 1.40)	0.03
Health anxiety	1.04 (1.01 to 1.07)	0.02	1.05 (1.01 to 1.07)	0.01	1.07 (1.03 to 1.11)	0.001	1.06 (1.02 to 1.09)	0.003

*Reference group.

FDR, first-degree relative; RA, rheumatoid arthritis.

With the exception of treatment control in the context of perceived comparative risk, the same three variables also remained in the final models for the other risk measures. Other variables that were associated with risk perception included increased perceived duration of RA (comparative and experiential risk), higher health literacy (experiential risk), female gender, higher perceived concern about RA and higher perceived negative emotional impact of RA (worry about Risk) (table 3).

A significant difference between children and siblings was found in all measures of perceived risk. Sensitivity analyses were conducted for children only as they constituted 75.4% of the FDR sample (online supplemental table 1). Univariate analyses demonstrated that age and the IPQ Personal Control subscale were significantly associated with perceived absolute risk for FDRs overall but not for children. Health anxiety and the IPQ Treatment Control subscale remained significantly associated in children. Interestingly, Health Literacy was significantly associated with perceived absolute risk in children but not FDRs overall. Sensitivity analyses for other measures of perceived risk is also shown in online supplemental table 1). Findings were similar with IPQ Treatment Control and Health Anxiety remaining significantly associated with perceived risk in children.

Patient variables and their associations with FDRs perceived absolute risk is summarised in table 4. Of the patient variables examined, only age predicted perceived experiential risk. No other patient variables were found to be predictors of perceived risk. Sensitivity analyses for children only can be found in online supplemental table 2. Patient age and age at diagnosis predicted children’s perceived experiential risk.

**Table 4:** Index patient descriptive statistics and GEEs

Index Patient Variable	All Patients n=213	FDRs’ perceived absolute risk				FDRs’ perceived comparative risk				FDRs’ perceived experiential risk				FDRs’ worry about risk			
		Low n=106	High n=184	W	P value	Low n=129	High n=161	W	P value	Low n=91	High n=200	W	P value	Low n=134	High n=157	W	P value
Age																	
	64 (55–73)	64 (56–70)	64 (57–73)	1.16	0.28	64 (54–70)	65 (58–73)	2.07	0.15	64 (54–70)	64 (57–73)	5.91	0.02	64 (55–71)	64 (58–70)	0.30	0.58
n=7 missing; median (IQR)																	
Gender																	
Male	50 (24.2)	18 (28.1)	46 (71.9)	2.38	0.12	28 (43.8)	36 (56.3)	0.08	0.78	17 (26.6)	47 (73.4)	0.73	0.39	32 (50.0)	32 (50.0)	0.46	0.50
Female	157 (75.8)	85 (38.8)	134 (61.2)			97 (44.3)	122 (55.7)			71 (32.3)	149 (67.7)			99 (45.0)	121 (55.0)		
n=6 missing; frequency (%)																	
Age at diagnosis																	
	52 (41–61)	50 (40–59)	53 (43–61)	1.97	0.16	52 (42–60)	52 (41–61)	0.08	0.78	50 (39–58)	54 (43–61)	3.05	0.08	52 (43–60)	52 (42–61)	0.01	0.94
n=47 missing; median (IQR)																	
RA treatment																	
Biologic	67 (31.5)	36 (38.3)	58 (61.7)	0.19	0.67	42 (44.7)	52 (55.3)	0.22	0.64	32 (34.0)	62 (66.0)	0.50	0.48	46 (48.9)	48 (51.1)	0.32	0.57
Non-biologic	146 (68.5)	70 (35.7)	126 (64.3)			87 (44.4)	109 (55.6)			59 (29.9)	138 (70.1)			88 (44.7)	109 (55.3)		
n=0 missing; frequency (%)																	
RAID Score																	
	4.9 (3–7)	5.0 (3–7)	4.9 (3–7)	0.00	0.96	5.0 (2–7)	4.9 (3–7)	0.09	0.76	4.6 (2–7)	5.0 (3–7)	0.55	0.46	4.7 (2–7)	5.0 (3 –7)	1.86	0.17

n=9 missing; Median (IQR).

213 patients and 291 FDRs.

FDR, first-degree relative; GEE, generalised estimating equation; RAID, Rheumatoid Arthritis Impact of Disease; W, Wald χ^2^.

## Discussion

This is the first study to quantify factors associated with perceived risk of RA in FDRs of patients with RA. The majority of FDRs perceived themselves to be at high absolute risk of RA, which supports previous qualitative results.^28^

FDRs have an approximately 3–5 fold increased risk of developing RA compared with the general population.^1^ Furthermore, there is an evidence to suggest that siblings of patients with RA have a higher incidence of developing RA compared with children.^29 30^ The results of this study suggest that siblings are less likely to perceive themselves at risk compared with children, therefore, highlighting the importance of effective risk communication in this susceptible group.

Lower age was associated with increased perceived risk among the whole cohort in univariate analysis but not in sensitivity analysis of children only or in the multivariate analysis. This suggests that it is being the child of a patient with RA which increases risk perception rather than age as an independent predictor. Children may feel more at risk than siblings because they may perceive that they have a greater period of time in which to develop RA. Siblings are likely to have lived longer without the disease, and therefore, may perceive themselves to be at less risk. It is also possible that children may feel more at risk as they likely had more first-hand experience of their proband’s RA while living in the same household during childhood. Comparatively, siblings are less likely to have cohabited with the index patient after the time of diagnosis, most commonly in adulthood. In this study, current cohabitation with the patient was assessed, and not found to be a predictor of perceived risk, but previous cohabitation and duration of cohabitation since the patient’s RA diagnosis were not explored.

Female sex was found to predict worry about risk but not perception of risk. While controlled for in the comparative risk measure, it is surprising that this well-established non-modifiable risk factor for the development of RA did not influence perceived absolute or experiential risk. This may indicate a lack of FDR knowledge about the disease and its risk factors which has consequently failed to translate in FDR risk perception. Further investigation of FDRs awareness of risk factors is needed.

Increased health anxiety scores were associated with higher perceived risk of RA. While this association has not been previously explored in the context of RA, studies in other disease have found similar associations.^31^ Importantly, in individuals with high health anxiety, despite perceiving themselves to be at high risk, there is evidence to suggest they may be less likely to engage in preventative strategies.^32^ Therefore, careful consideration about the strategies implemented to communicate risk is required. Similarly, effective communication is also required for those with low health anxiety who may not perceive themselves to be at risk.

Lower perceptions of how successful treatment would be at controlling RA, as measured by the brief IPQ, was associated with higher perceived risk. This aligns with the results from a cohort of individuals at risk of venous thrombosis where increased perception of how successful a treatment for thrombosis significantly predicted lower perceived risk of thrombosis.^33^ Conversely, in individuals with familial hypercholesterolaemia there was not a significant association found between the efficacy of medication or lifestyle changes in reducing CVD risk and perceived risk.^34^ In addition, it has been shown that the manner in which treatment risks and benefits are presented can influence health decisions.^35^ This highlights the importance of treatment efficacy education when communicating risk to FDRs.

Patient age was associated with FDRs perceived experiential risk, but no other risk perception measures, and there were no significant associations between other patient characteristics and risk perception. Considering the potential for type 1 errors there was therefore no evidence of an association between FDRs perceived risk and variables relating to their index patient.

### Implications

Clinicians can experience difficulty in interpretation and effective communication of possible risk^36^ and, concurrent to this, patients may encounter challenges in understanding and accurately assessing their own risk.^37^ Risk conveyance is most effective when tailored to an individual’s characteristics^38^ and personalised risk information can increase an individual’s motivation to improve risk-related behaviours.^39^

European Alliance of Associations for Rheumatology guidelines for trials of preventive interventions in at-risk groups emphasise the importance of developing effective, tailored risk communication tools for RA.^4^ The findings of this study increase understanding of the factors that can influence FDRs’ perceived risk of developing RA with implications for the content of material to support discussions related to risk in this at-risk population.

### Strengths and limitations

The methodological strengths of this study include a large sample size of both FDRs and index patients, paired data linking FDRs with index patients and statistical analysis that accounted for possible non-independence of multiple FDRs linked to the same index patient. While there were many linked pairs, it was not uncommon for an FDR to complete the survey when their respective index patient did not. Possible reasons for this are that (1i) FDRs may feel they have more of a vested interest in the research topic/more to gain from the outcome of this study and subsequent research related to prediction and prevention of RA. (2) The FDR survey was shorter than the one that patients were asked to complete (the latter including items exploring their likelihood of communicating with relatives about risk of RA). (3) Family communication about risk is complex, and may be associated with feelings of guilt and anxiety as highlighted in previous qualitative research.^40^ Therefore, this may have led to fewer patients completing and returning the survey.

Input from PRPs and multidisciplinary contributors during protocol and survey design enhanced survey design and mitigated for potential anxiety that participants may have felt when considering their risk, or their relatives’ risk of developing RA.

FDRs were recruited through patients with a confirmed diagnosis of RA rather than relying on self-reported family history of RA. Therefore, the chance of incorrect participant enrolment related to confusion between RA and other conditions such as osteoarthritis^41^ was minimised. It did, however, increase the chance of selection bias as FDR recruitment was reliant on patients distributing the survey, and therefore, the characteristics of non-responder patients/FDRs and FDRs that were not invited by an index patient are not represented in this sample. It is also possible that FDRs with higher health anxiety or higher risk perception were more likely to participate in the study. This may have introduced further selection bias and overestimation of the perceived risk of FDRs. This highlights the challenges of FDR recruitment^42^ and the need for consideration of alternative recruitment strategies.

A possible predictor of risk perception that was not accounted for in this study is the FDR’s total number of relatives with RA. An increased number of affected relatives or the characteristics of another affected relative who was not the index patient may have influenced FDR risk perception. The impact of a broader family history on risk perception in RA will be important to explore in future research as such an impact has been demonstrated in other diseases.^43 44^

Fewer respondents perceived themselves to be at low perceived risk than was expected and subsequently, the very low, low and neutral perceived risk groups were combined when the outcome variables were dichotomised. While this approach allowed for appropriate statistical analyses, the differences between each of these individual groups were not evaluated in this study.

The smoking status of FDRs was not assessed in this study. Tobacco smoking is a recognised modifiable risk factor^5^ and it is possible that FDRs who smoke tobacco may have a different perception of risk to a non-smoker, which was not captured in this analysis. As previously noted, further investigation of the awareness of risk factors for RA among FDRs is needed.

The serological status of index patients was not assessed. Seropositivity has been associated with poorer functional ability.^45^ It is therefore plausible that serological status may impact FDR perception of risk.

Finally, disease activity of the index patient during the previous week was assessed using the RAID score in the patient survey. Against expectations this score was not associated with the level of perceived risk. It is possible that both objective and subjective measures of disease impact and severity over the duration of the diagnosis would have had greater influence on FDRs’ perceived risk.

## Conclusion

Among FDRs, perceived risk of RA was high. Three main predictors of perceived risk were identified which were being a child of a patient with RA, higher health anxiety and lower perceptions of treatment control. Understanding of these predictors will inform the development of effective risk communication strategies and aid RA prevention and early intervention efforts.

## Data Availability

Data are available on reasonable request. Data available from the authors on reasonable request.

## References

[1] Symmons D, Turner G, Webb R, et al. The prevalence of rheumatoid arthritis in the United Kingdom: new estimates for a new century. Rheumatology 2002;41:793–800. 10.1093/rheumatology/41.7.793 12096230

[2] Hsieh P-H, Wu O, Geue C, et al. Economic burden of rheumatoid arthritis: a systematic review of literature in biologic era. Ann Rheum Dis 2020;79:771–7. 10.1136/annrheumdis-2019-216243 32245893

[3] Mankia K, Siddle H, Di Matteo A, et al. A core set of risk factors in individuals at risk of rheumatoid arthritis: a systematic literature review Informing the EULAR points to consider for conducting clinical trials and observational studies in individuals at risk of rheumatoid arthritis. RMD Open 2021;7:e001768. 10.1136/rmdopen-2021-001768 34531306 PMC8449955

[4] Mankia K, Siddle HJ, Kerschbaumer A, et al. EULAR points to consider for conducting clinical trials and observational studies in individuals at risk of rheumatoid arthritis. Ann Rheum Dis 2021;80:1286–98. 10.1136/annrheumdis-2021-220884 34362746 PMC8458095

[5] Romão VC, Fonseca JE. Etiology and risk factors for rheumatoid arthritis: a state-of-the-art review. Front Med 2021;8:689698. 10.3389/fmed.2021.689698 PMC8661097 34901047

[6] Raza K, Klareskog L, Holers VM. Predicting and preventing the development of rheumatoid arthritis. Rheumatology 2016;55:1–3. 10.1093/rheumatology/kev261 26224307 PMC5854029

[7] Wells I, Zemedikun DT, Simons G, et al. Predictors of interest in predictive testing for rheumatoid arthritis among first degree relatives of rheumatoid arthritis patients. Rheumatology 2021. 10.1093/rheumatology/keab890 PMC9348622 34850849

[8] Simons G, Stack RJ, Stoffer-Marx M, et al. Perceptions of first-degree relatives of patients with rheumatoid arthritis about lifestyle modifications and pharmacological interventions to reduce the risk of rheumatoid arthritis development: a qualitative interview study. BMC Rheumatol 2018;2:31. 10.1186/s41927-018-0038-3 30886981 PMC6390593

[9] Ferrer R, Klein WM. Risk perceptions and health behavior. Curr Opin Psychol 2015;5:85–9. 10.1016/j.copsyc.2015.03.012 26258160 PMC4525709

[10] Becker M. The health belief model and personal health behavior. Health Educ Monogr 1974:324–508.

[11] Imes CC, Novosel LM, Burke LE. Heart disease risk and self-efficacy in overweight and obese adults. J Nurse Pract 2016;12:710–6. 10.1016/j.nurpra.2016.09.008 28408861 PMC5385996

[12] Haug U, Riedel O, Cholmakow-Bodechtel C, et al. First-degree relatives of cancer patients: a target group for primary prevention? A cross-sectional study. Br J Cancer 2018;118:1255–61. 10.1038/s41416-018-0057-2 29559731 PMC5943415

[13] Beebe-Dimmer JL, Wood DP, Gruber SB, et al. Risk perception and concern among brothers of men with prostate carcinoma. Cancer 2004;100:1537–44. 10.1002/cncr.20121 15042690

[14] van Esch SCM, Nijkamp MD, Cornel MC, et al. Illness representations of type 2 diabetes patients are associated with perceptions of diabetes threat in relatives. J Health Psychol 2014;19:358–68. 10.1177/1359105312470853 23399919

[15] Whitney CA, Dorfman CS, Shelby RA, et al. Reminders of cancer risk and pain catastrophizing: relationships with cancer worry and perceived risk in women with a first-degree relative with breast cancer. Fam Cancer 2019;18:9–18. 10.1007/s10689-018-0082-6 29679190 PMC7028392

[16] Klein WMP. Self-prescriptive, perceived, and actual attention to comparative risk information. Psychol Health 2003;18:625–43. 10.1080/0887044031000118465

[17] Dillard AJ, Ferrer RA, Ubel PA, et al. Risk perception measures’ associations with behavior intentions, affect, and cognition following colon cancer screening messages. Health Psychology 2012;31:106–13. 10.1037/a0024787 21806302

[18] Weinstein ND, Kwitel A, McCaul KD, et al. Risk perceptions: assessment and relationship to influenza vaccination. Health Psychol 2007;26:146–51. 10.1037/0278-6133.26.2.146 17385965

[19] Sjöberg L. Worry and risk perception. Risk Analysis 1998;18:85–93. 10.1111/j.1539-6924.1998.tb00918.x 9523446

[20] Broadbent E, Petrie KJ, Main J, et al. The brief illness perception questionnaire. J Psychosom Res 2006;60:631–7. 10.1016/j.jpsychores.2005.10.020 16731240

[21] Figueiras MJ, Alves NC. Lay perceptions of serious illnesses: an adapted version of the revised illness perception questionnaire (IPQ-R) for healthy people. Psychol Health 2007;22:143–58. 10.1080/14768320600774462

[22] McNaughton CD, Cavanaugh KL, Kripalani S, et al. Validation of a short, 3-Item version of the subjective numeracy scale. Med Decis Making 2015;35:932–6. 10.1177/0272989X15581800 25878195 PMC4592371

[23] Morris NS, MacLean CD, Chew LD, et al. The single item literacy screener: evaluation of a brief instrument to identify limited reading ability. BMC Fam Pract 2006;7:21. 10.1186/1471-2296-7-21 16563164 PMC1435902

[24] Finset A, Steine S, Haugli L, et al. The brief approach/avoidance coping questionnaire: development and validation. Psychol Health Med 2002;7:75–85. 10.1080/13548500120101577

[25] Scheier MF, Carver CS, Bridges MW. Distinguishing optimism from neuroticism (and trait anxiety, self-mastery, and self-esteem): a reevaluation of the life orientation test. J Pers Soc Psychol 1994;67:1063–78. 10.1037/0022-3514.67.6.1063 7815302

[26] Salkovskis PM, Rimes KA, Warwick HMC, et al. The health anxiety inventory: development and validation of scales for the measurement of health anxiety and hypochondriasis. Psychol Med 2002;32:843–53. 10.1017/S0033291702005822 12171378

[27] Gossec L, Dougados M, Rincheval N, et al. Elaboration of the preliminary rheumatoid arthritis impact of disease (raid) score: a EULAR initiative. Ann Rheum Dis 2009;68:1680–5. 10.1136/ard.2008.100271 19054825

[28] Stack RJ, Stoffer M, Englbrecht M, et al. Perceptions of risk and predictive testing held by the first-degree relatives of patients with rheumatoid arthritis in England, Austria and Germany: a qualitative study. BMJ Open 2016;6:e010555. 10.1136/bmjopen-2015-010555 PMC4932277 27357193

[29] Hemminki K, Li X, Sundquist J, et al. Familial associations of rheumatoid arthritis with autoimmune diseases and related conditions. Arthritis Rheum 2009;60:661–8. 10.1002/art.24328 19248111

[30] Kuo C-F, Grainge MJ, Valdes AM, et al. Familial aggregation of rheumatoid arthritis and co-aggregation of autoimmune diseases in affected families: a nationwide population-based study. Rheumatology 2017;56:928–33. 10.1093/rheumatology/kew500 28160009 PMC5850742

[31] Godino JG, van Sluijs EMF, Sutton S, et al. Understanding perceived risk of type 2 diabetes in healthy middle-aged adults: a cross-sectional study of associations with modelled risk, clinical risk factors, and psychological factors. Diabetes Res Clin Pract 2014;106:412–9. 10.1016/j.diabres.2014.10.004 25467619 PMC4337811

[32] Hadjistavropoulos HD, Craig KD, Hadjistavropoulos T. Cognitive and behavioral responses to illness information: the role of health anxiety. Behav Res Ther 1998;36:149–64. 10.1016/S0005-7967(98)00014-X 9613022

[33] Korlaar IV, Vossen C, Rosendaal FR. Using the common-sense model to predict risk perception and disease- related worry in individuals at increased risk for venous thrombosis, 2007. 10.1037/0278-6133.26.6.807 18020855

[34] Claassen L, Henneman L, Kindt I, et al. Perceived risk and representations of cardiovascular disease and preventive behaviour in people diagnosed with familial hypercholesterolemia: a cross-sectional questionnaire study. J Health Psychol 2010;15:33–43. 10.1177/1359105309345170 20064882

[35] Carling CLL, Kristoffersen DT, Montori VM, et al. The effect of alternative summary statistics for communicating risk reduction on decisions about taking statins: a randomized trial. PLoS Med 2009;6:e1000134. 10.1371/journal.pmed.1000134 19707575 PMC2724738

[36] Gigerenzer G, Gaissmaier W, Kurz-Milcke E, et al. Helping doctors and patients make sense of health statistics. Psychol Sci Public Interest 2007;8:53–96. 10.1111/j.1539-6053.2008.00033.x 26161749

[37] Smerecnik CMR, Mesters I, Verweij E, et al. A systematic review of the impact of genetic counseling on risk perception accuracy. J Genet Couns 2009;18:217–28. 10.1007/s10897-008-9210-z 19291376 PMC7451018

[38] Peters E, Hart PS, Fraenkel L. Informing patients: the influence of numeracy, framing, and format of side effect information on risk perceptions. Med Decis Making 2011;31:432–6. 10.1177/0272989X10391672 21191122

[39] Sparks JA, Iversen MD, Yu Z, et al. Disclosure of personalized rheumatoid arthritis risk using genetics, biomarkers, and lifestyle factors to Motivate health behavior improvements: a randomized controlled trial. Arthritis Care Res 2018;70:823–33. 10.1002/acr.23411 PMC5897224 29024454

[40] Falahee M, Simons G, Buckley CD, et al. Patients’ perceptions of their relatives’ risk of developing rheumatoid arthritis and of the potential for risk communication, prediction, and modulation. Arthritis Care Res 2017;69:1558–65. 10.1002/acr.23179 27998043

[41] Simons G, Mason A, Falahee M, et al. Qualitative exploration of illness perceptions of rheumatoid arthritis in the general public. Musculoskeletal Care 2017;15:13–22. 10.1002/msc.1135 26833593 PMC4903170

[42] van Boheemen L, Ter Wee MM, Seppen B, et al. How to enhance recruitment of individuals at risk of rheumatoid arthritis into trials aimed at prevention: understanding the barriers and facilitators. RMD Open 2021;7:e001592. 10.1136/rmdopen-2021-001592 33685929 PMC7942248

[43] Davids SL, Schapira MM, McAuliffe TL, et al. Predictors of pessimistic breast cancer risk perceptions in a primary care population. J Gen Intern Med 2004;19:310–5. 10.1111/j.1525-1497.2004.20801.x 15061739 PMC1492192

[44] Bratt O, Damber JE, Emanuelsson M, et al. Risk perception, screening practice and interest in genetic testing among unaffected men in families with hereditary prostate cancer. Eur J Cancer 2000;36:235–41. 10.1016/S0959-8049(99)00272-5 10741283

[45] Graell E, Vazquez I, Larrosa M, et al. Disability measured by the modified health assessment questionnaire in early rheumatoid arthritis: prognostic factors after two years of follow-up. Clin Exp Rheumatol 2009;27:284–91. 19473570

