# Supplementary material for: Predictors of perceived risk in first-degree relatives of patients with rheumatoid arthritis
Source: RMD Open. 2022 Dec 5;8(2):e002606. doi: 10.1136/rmdopen-2022-002606 (PMC9723950; doi:10.1136/rmdopen-2022-002606)
Supplement: Supplementary data [file rmdopen-2022-002606supp001.pdf]

# Supplementary Materials

**Figure 1:** Scatterplots and Spearman Correlations for Risk Measures

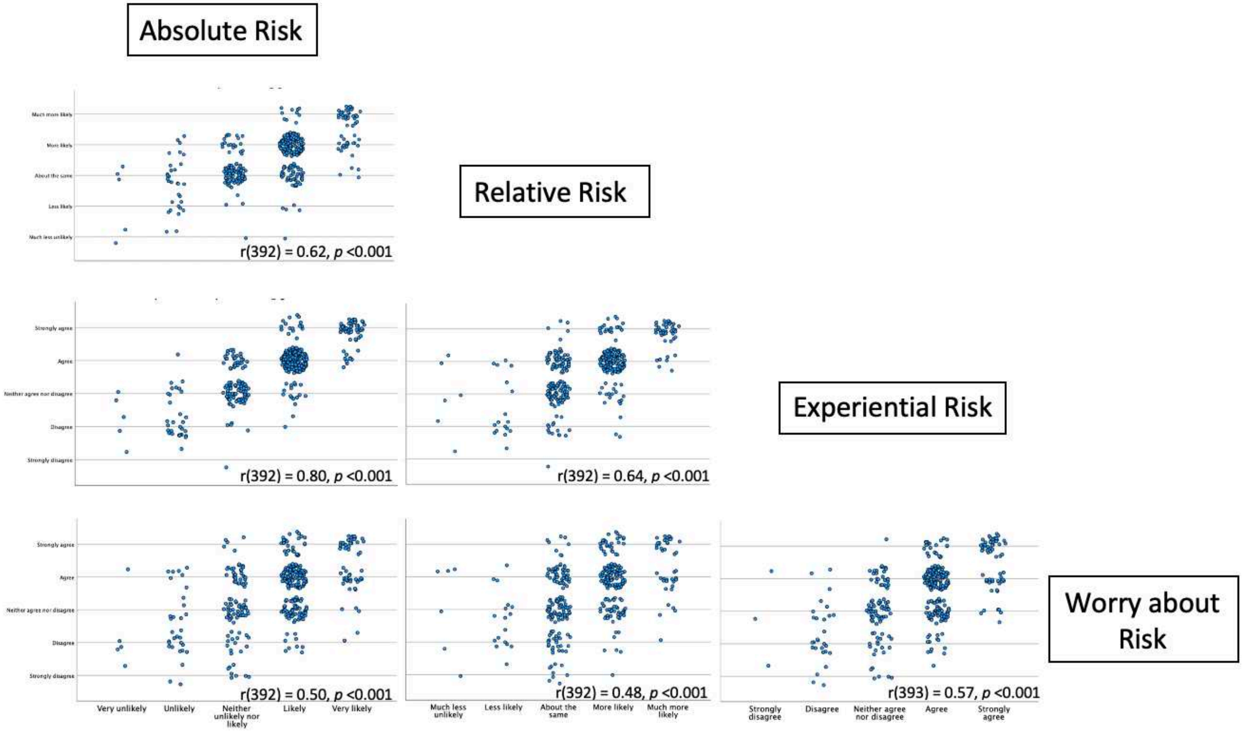

**Table 1:** Sensitivity analysis (children only) – Univariate Analysis

| **Characteristic of FDR** | **All Children** *(n=295)* | **Sensitivity Analysis – Children Only** | | | | | | | | | | | |
|---|---|---|---|---|---|---|---|---|---|---|---|---|---|
| | | **Perceived Absolute Risk** | | | **Perceived Relative Risk** | | | **Perceived Experiential Risk** | | | **Worry about Risk** | | |
| | | **Low** *(n=86)* | **High** *(n=208)* | ***p*-value** | **Low** *(n=113)* | **High** *(n=181)* | ***p*-value** | **Low** *(n=72)* | **High** *(n=223)* | ***p*-value** | **Low** *(n=115)* | **High** *(n=180)* | ***p*-value** |
| **Age** (years) *n = 16 missing* *Mean (SD); T* | 38.1 (11.8) | 37.1 (11.9) | 38.6 (11.8) | 0.34 | 37.5 (12.8) | 38.6 (11.3) | 0.46 | 37.7 (12.1) | 38.3 (11.3) | 0.73 | 37.4 (12.0) | 38.6 (11.7) | 0.42 |
| **Gender** | | | | | | | | | | | | | |
| Male | 107 (36.9) | 34 (31.8) | 73 (68.2) | 0.50 | 47 (43.9) | 60 (56.1) | 0.14 | 32 (29.9) | 75 (70.1) | 0.10 | 56 (52.3) | 51 (47.7) | **<0.001*** |
| Female | 183 (63.1) | 51 (28.0) | 131 (72.0) | | 64 (35.2) | 118 (64.8) | | 39 (21.3) | 144 (78.7) | | 57 (31.1) | 126 (68.9) | |
| *n= 6 missing* *Frequency (%); CS* | | | | | | | | | | | | | |
| **Ethnic Group** | | | | | | | | | | | | | |
| White | 241 (82.3) | 68 (28.2) | 173 (71.8) | 0.25 | 94 (39.0) | 147 (61.0) | 0.77 | 58 (24.1) | 183 (75.9) | 0.93 | 94 (39.0) | 147 (61.0) | 0.47 |
| Asian | 27 (9.2) | 9 (33.3) | 18 (66.7) | | 8 (29.6) | 19 (70.4) | | 7 (25.9) | 20 (74.1) | | 8 (29.4) | 19 (70.4) | |
| Black | 12 (4.1) | 1 (9.1) | 10 (90.9) | | 5 (45.5) | 6 (54.5) | | 3 (25.0) | 9 (75.0) | | 7 (58.3) | 5 (41.7) | |
| Mixed | 12 (4.1) | 6 (50.0) | 6 (50.0) | | 5 (41.7) | 7 (58.3) | | 4 (33.3) | 8 (66.7) | | 5 (41.7) | 7 (58.3) | |
| Other | 1 (0.3) | 0 (0.0) | 1 (100.0) | | 0 (0.0) | 1 (100.0) | | 0 (0.0) | 1 (100.0) | | 0 (0.0) | 1 (100.0) | |
| *n=2 missing* *Frequency (%); CS* | | | | | | | | | | | | | |
| **Deprivation Index** *n=82 missing* *Median (IQR); U* | 4 (2-7) | 5 (2-7) | 4 (2-7) | 0.33 | 4 (2-7) | 4.5 (2-7) | 0.45 | 6 (3-7.5) | 4 (2-7) | 0.15 | 5 (2-7) | 4 (2-7) | 0.35 |
| **Employment Status** | | | | | | | | | | | | | |
| Employed | 240 (82.9) | 75 (31.3) | 165 (68.8) | 0.22 | 98 (40.8) | 142 (59.2) | 0.24 | 60 (25.0) | 180 (75.0) | 0.32 | 96 (40.0) | 144 (60.0) | 0.14 |
| Unemployed | 22 (7.6) | 3 (13.6) | 19 (86.4) | | 5 (22.7) | 17 (77.3) | | 3 (13.6) | 19 (86.4) | | 5 (22.7) | 17 (77.3) | |
| Other | 28 (9.7) | 8 (29.6) | 19 (70.4) | | 10 (37.0) | 17 (63.0) | | 9 (32.1) | 19 (67.9) | | 14 (50.0) | 14 (50.0) | |
| *n=6 missing* *Frequency (%); CS* | | | | | | | | | | | | | |
| **Education Level** | | | | | | | | | | | | | |
| A-level or lower | 134 (47.2) | 40 (30.1) | 93 (69.9) | 0.99 | 52 (39.1) | 81 (60.9) | 0.88 | 39 (29.1) | 95 (70.9) | 0.17 | 48 (35.8) | 86 (64.2) | 0.20 |
| Higher than A-Level | 150 (52.8) | 45 (30.0) | 105 (70.0) | | 60 (40.0) | 90 (60.0) | | 33 (22.0) | 117 (78.0) | | 65 (43.3) | 85 (56.7) | |
| *n=17 missing* *Frequency (%); CS* | | | | | | | | | | | | | |
| **Smoking Status** | | | | | | | | | | | | | |
| Current smoker | 34 (11.8) | 9 (26.5) | 25 (73.5) | 0.74 | 10 (29.4) | 24 (70.6) | 0.39 | 9 (26.5) | 25 (73.5) | 0.92 | 13 (38.2) | 21 (61.8) | 0.57 |
| Ex-smoker | 80 (27.8) | 22 (27.8) | 57 (72.2) | | 34 (43.0) | 45 (57.0) | | 21 (26.3) | 59 (73.8) | | 28 (35.0) | 52 (65.0) | |
| Never smoked | 174 (60.4) | 55 (31.6) | 119 (68.4) | | 69 (39.7) | 105 (60.3) | | 42 (24.1) | 132 (75.9) | | 73 (42.0) | 101 (58.0) | |
| *n=8 missing* *Frequency (%); CS* | | | | | | | | | | | | | |
| **Relationship to index patient** | | | | | | | | | | | | | |
| Child | 295 (100.0) | 86 (29.3) | 208 (70.7) | - | 113 (38.4) | 181 (61.6) | - | 72 (24.4) | 223 (75.6) | - | 115 (39.0) | 180 (61.0) | - |
| Sibling | - | - | - | | - | - | | - | - | | - | - | |
| *n=4 missing* *Frequency (%); CS* | | | | | | | | | | | | | |
| **Living in same household as index patient** | | | | | | | | | | | | | |

| | | | | | | | | | | | | | |
|---|---|---|---|---|---|---|---|---|---|---|---|---|---|
| Yes | 73 (24.8) | 24 (32.9) | 49 (67.1) | 0.40 | 35 (47.9) | 38 (52.1) | 0.05 | 19 (26.0) | 54 (74.0) | 0.67 | 34 (46.6) | 39 (53.4) | 0.12 |
| No | 221 (75.2) | 61 (27.7) | 159 (72.3) | | 77 (35.0) | 143 (65.0) | | 52 (23.5) | 169 (76.5) | | 80 (36.2) | 141 (63.8) | |
| *n=2 missing* *Frequency (%); CS* | | | | | | | | | | | | | |
| **Frequency of communication with index patient** | | | | | | | | | | | | | |
| Never | 0 (0.0) | 0 (0.0) | 0 (0.0) | 0.39 | 0 (0.0) | 0 (0.0) | 0.72 | 0 (0.0) | 0 (0.0) | 0.82 | 0 (0.0) | 0 (0.0) | 0.06 |
| Rarely | 0 (0.0) | 0 (0.0) | 0 (0.0) | | 0 (0.0) | 0 (0.0) | | 0 (0.0) | 0 (0.0) | | 0 (0.0) | 0 (0.0) | |
| Sometimes | 8 (2.7) | 4 (50.0) | 4 (50.0) | | 3 (37.5) | 5 (62.5) | | 2 (25.0) | 6 (75.0) | | 4 (50.0) | 4 (50.0) | |
| Often | 96 (32.8) | 26 (27.1) | 70 (72.9) | | 40 (41.7) | 56 (58.3) | | 24 (25.0) | 72 (75.0) | | 44 (45.6) | 52 (54.2) | |
| Daily | 189 (64.5) | 55 (29.3) | 133 (70.7) | | 69 (36.7) | 119 (63.3) | | 45 (23.8) | 144 (76.2) | | 66 (34.9) | 123 (65.1) | |
| *n=4 missing* *Frequency (%); U* | | | | | | | | | | | | | |
| **Subjective Numeracy** *n=4 missing* *Median (IQR); U* | 15 (12-17) | 15 (13 - 17.5) | 15 (12-18) | 0.30 | 15 (12-17) | 15 (12-18) | 0.66 | 15 (13-17) | 15 (12-18) | 0.41 | 16 (13-18) | 14 (11-17.5) | **0.01*** |
| **Health Literacy** *n=4 missing* *Median (IQR); U* | 0 (0-0) | 0 (0-0) | 0 (0-0) | **0.01*** | 0 (0-0) | 0 (0-0) | 0.11 | 0 (0-0) | 0 (0-0) | **0.007*** | 0 (0-0) | 0 (0-0) | 0.92 |
| | | | | | | | | | | | | | |
| **Illness Perceptions** | | | | | | | | | | | | | |
| Consequences *(n=4 missing)* | 9 (7-9) | 8 (7-9) | 8 (7-9) | 0.35 | 8 (7-9) | 8 (7-9) | 0.18 | 8 (7-9) | 8 (7-9) | 0.09 | 7 (6-8) | 8 (7-9) | **<0.001*** |
| Timeline *(n=5 missing)* | 10 (9-10) | 10 (8-10) | 10 (9-10) | 0.09 | 10 (8-10) | 10 (9.25-10) | **0.002*** | 10 (8-10) | 10 (9-10) | 0.05 | 10 (8-10) | 10 (9-10) | **0.008*** |
| Personal control *(n=5 missing)* | 5 (3-6) | 5 (3-6) | 5 (3-6) | 0.90 | 5 (3-6) | 5 (3-6) | 0.71 | 5 (3-6) | 5 (3-6) | 0.64 | 5 (3-6) | 5 (3-6) | 0.45 |
| Treatment control *(n=5 missing)* | 7 (5-7) | 7 (5-8) | 6 (5-8) | **0.03*** | 7 (5-8) | 6 (5-7) | **0.02*** | 7 (5-8.5) | 6 (5-8) | **0.03*** | 7 (5-8) | 6 (5-7) | **0.001*** |
| Identity *(n= 4 missing)* | 8 (7-8) | 8 (6.5-8) | 7 (7-8) | 0.41 | 7 (6-8) | 8 (7-8) | 0.07 | 8 (6-8) | 7 (7-8) | 0.26 | 7 (6-8) | 8 (7-8.5) | **<0.001*** |
| Concern *(n=2 missing)* | 8 (7-10) | 8 (7-10) | 8 (7-10) | 0.16 | 8 (7-10) | 8 (7-10) | 0.17 | 8 (7-10) | 8 (7-10) | 0.09 | 8 (6-8) | 9 (8-10) | **<0.001*** |
| Understanding *(n=2 missing)* | 7 (6-9) | 7 (6-9) | 7 (6-9) | 0.36 | 7 (6-9) | 7 (6-9) | 0.45 | 7 (6-9) | 7 (6-9) | 0.42 | 8 (6-9) | 7 (6-9) | 0.27 |
| Emotion *(n=2 missing)* *Median (IQR); U* | 7 (6-9) | 7 (6-9) | 7 (6-9) | 0.61 | 7 (6-9) | 7 (6-9) | 0.23 | 7 (5-8) | 7 (6-9) | **0.01*** | 7 (5-7) | 8 (7-9) | **<0.001*** |
| **Coping Style** *n = 8 missing* *Mean (SD); T* | 29.8 (5.7) | 29.4 (5.9) | 29.9 (5.9) | 0.58 | 29.7 (5.9) | 29.8 (5.6) | 0.89 | 30.0 (6.1) | 29.7 (5.6) | 0.73 | 29.8 (6.2) | 29.7 (5.4) | 0.93 |
| **Optimism** *n = 5 missing* *Mean (SD); T* | 7.1 (2.4) | 7.1 (2.5) | 7.2 (2.4) | 0.86 | 7.3 (2.6) | 7.1 (2.3) | 0.48 | 7.3 (2.4) | 7.1 (2.4) | 0.62 | 7.1 (2.8) | 7.2 (2.3) | 0.71 |
| **Health Anxiety** *n = 17 missing* *Median (IQR); U* | 12 (8-18) | 11 (7-15) | 13 (8-19) | **0.046*** | 11 (7-16) | 13 (9-19) | **0.001*** | 11 (6-13.5) | 13 (9-19) | **<0.001*** | 9 (6-14) | 14 (9-20) | **<0.001*** |

T = Independent samples T-test, CS = Chi-Square test, U = Mann-Whitney U Test

**Table 2:** Sensitivity Analysis (children only) - Index Patient Descriptive Statistics and GEEs (Relative Risk, Experiential Risk, Worry about Risk)

| **Characteristic of Index Patient** | **All Patients** *(n=162 patients)* | **GEEs – Sensitivity Analysis – Children Only** | | | | | | | | | | | | | | | |
|---|---|---|---|---|---|---|---|---|---|---|---|---|---|---|---|---|---|
| | | **Perceived Absolute Risk** | | | | **Perceived Relative Risk** | | | | **Perceived Experiential Risk** | | | | **Worry about Risk** | | | |
| | | **Low** *(n=74 FDRs)* | **High** *(n=107 FDRs)* | **WCS** | ***p-value*** | **Low** *(n=90 FDRs)* | **High** *(n=131 FDRs)* | **WCS** | ***p-value*** | **Low** *(n=60 FDRs)* | **High** *(n=162 FDRs)* | **WCS** | ***p-value*** | **Low** *(n=96 FDRs)* | **High** *(n=126 FDRs)* | **WCS** | ***p-value*** |
| **Age** (years) *n = 7 missing Median (IQR)* | 65 (57.25-75) | 64 (57.5-70.5) | 66 (58.5-75.0) | 2.40 | 0.12 | 64 (57.5-70.5) | 66 (58.5-75.0) | 1.58 | 0.21 | 64 (54.75-70) | 65 (58.5-75.0) | 4.41 | **0.04*** | 64 (57.5-75) | 65 (59-74.25) | 0.13 | 0.71 |
| **Gender** | | | | | | | | | | | | | | | | | |
| Male | 40 (24.7) | 13 (24.5) | 40 (75.5) | 2.27 | 0.13 | 13 (24.5) | 40 (75.5) | 0.03 | 0.88 | 11 (20.8) | 42 (79.2) | 1.13 | 0.29 | 25 (47.2) | 28 (52.8) | 0.26 | 0.61 |
| Female *n= 6 missing Frequency (%)* | 117 (72.2) | 59 (36.2) | 104 (63.8) | | | 59 (36.2) | 104 (63.8) | | | 47 (28.7) | 117 (71.3) | | | 69 (42.1) | 95 (57.9) | | |
| **Age at diagnosis** *n = 47 missing Median (IQR)* | 54 (43.5-62) | 50 (41.5-60.0) | 54 (47-63) | 3.76 | 0.05 | 50 (41.5-60.0) | 54 (43-63) | 0.18 | 0.67 | 50 (43-58.5) | 54 (46-63) | 4.26 | **0.04*** | 53 (44.5-62.5) | 54 (43-61) | 0.45 | 0.51 |
| **RA Treatment** | | | | | | | | | | | | | | | | | |
| Biologic | 47 (29.0) | 20 (31.3) | 44 (68.8) | 0.17 | 0.68 | 20 (31.3) | 44 (68.8) | 0.42 | 0.52 | 17 (26.6) | 47 (73.4) | 0.01 | 0.94 | 31 (48.4) | 33 (51.6) | 0.64 | 0.43 |
| Non-Biologic *n = 0 missing Frequency (%)* | 115(71.0) | 54 (34.4) | 103 (65.6) | | | 54 (34.4) | 103 (65.6) | | | 43 (27.2) | 115 (72.8) | | | 65 (41.1) | 93 (58.9) | | |

GEE = Generalised Estimating Equation, WCS = Wald Chi-Square
